# Primary large cell neuroendocrine carcinoma of the breast with metastasis in the lymph nodes: a case report and literature review

**DOI:** 10.3389/fonc.2026.1812230

**Published:** 2026-06-11

**Authors:** HongYan Li, MaoTao He, HengMing Zhang, ChunYu Yu, MaiQing Yang

**Affiliations:** 1 Department of Outpatient, Weifang People’s Hospital (First Affiliated Hospital of Shandong Second Medical University), Weifang, Shandong, China; 2 Department of Pathology, Weifang People’s Hospital (First Affiliated Hospital of Shandong Second Medical University), Weifang, Shandong, China; 3 Department of Pathology and Pathophysiology, School of Basic Medical Sciences, Shandong Second Medical University, Weifang, Shandong, China

**Keywords:** breast, diagnosis, immunohistochemical, large-cell neuroendocrine carcinoma, misdiagnosis

## Abstract

Primary large cell neuroendocrine carcinoma (LCNEC) of the breast is an extremely rare and diagnostically challenging subtype. A 47-year-old female was admitted to Weifang People’s Hospital (Weifang, China) with a left breast mass that was incidentally detected. The tumor demonstrated a unique triple−negative immunophenotype and presented significant diagnostic pitfalls, mimicking conventional triple−negative breast cancer (TNBC). Modified radical mastectomy was performed after neoadjuvant chemotherapy. Based on the clinical, imaging and pathological findings, she was diagnosed with primary LCNEC of the breast with lymph node metastasis. Accurate distinction from TNBC with focal neuroendocrine differentiation and metastatic neuroendocrine carcinoma is critical. Short-term follow-up (9 months) suggests favorable disease control, but long-term efficacy and prognosis remain undetermined due to limited follow-up. This case highlights the clinical significance of avoiding misdiagnosis, with immunohistochemistry providing key diagnostic clues.

## 1 Introduction

Breast cancer is a heterogeneous group of malignancies with diverse histological subtypes, clinical behaviors, and molecular characteristics, which significantly influence treatment strategies and patient outcomes (1, 2). Among these subtypes, neuroendocrine carcinomas (NECs) of the breast represent an extremely rare and distinct entity, accounting for less than 5% of all primary breast carcinomas (3–5). Primary large cell neuroendocrine carcinoma (LCNEC) of the breast, a specific subtype of breast NECs, is even more uncommon, with fewer than 10 well-documented cases reported in the English literature and small retrospective studies documented in the medical literature to date (3, 6), leading to a paucity of comprehensive understanding regarding its pathogenesis, clinical manifestations, and optimal management. The diagnostic criteria for breast LCNEC are as follows: (1) a primary breast carcinoma exhibiting large cell neuroendocrine morphology, (2) diffuse or focal expression of at least one neuroendocrine marker, (3) exclusion of metastatic neuroendocrine carcinoma from other organs, and (4) high-grade histology with marked nuclear atypia and high proliferative activity (7, 8). Given its rarity and nonspecific clinical and radiological features, primary breast LCNEC is often misdiagnosed as invasive ductal carcinoma or other triple-negative breast cancer (TNBC) subtypes. Accurate diagnosis relies on careful morphological assessment combined with a comprehensive immunohistochemical panel. This paper presents a case of breast LCNEC accompanied by lymph node metastasis, with the purpose of enhancing the clinical and pathological understanding of this rare disease.

## 2 Case presentation

### 2.1 Clinical history

A 47-year-old female patient was admitted to our hospital for further treatment, presenting with a left breast mass that was incidentally detected 3 days before. The patient’s general state was normal. On palpation, a mass was felt in the left breast. Magnetic resonance imaging (MRI) showed an irregular mass in the upper outer quadrant of the left breast with a rough margin. The size of the lesion was approximately 1.5 cm × 1.7 cm × 2.1 cm (**Figure 1A**). Slightly enlarged lymph nodes were visible in the left axilla. A breast biopsy was performed, and the pathological result was invasive breast carcinoma, not otherwise specified (NOS). Systemic imaging workup, including chest computed tomography (CT), abdominal/pelvic CT, and whole-body positron emission tomography (PET)-CT, revealed no evidence of extra-mammary primary tumors or distant metastasis. Based on the immunohistochemical findings, the patient underwent radical mastectomy after neoadjuvant therapy.

**Figure 1:**
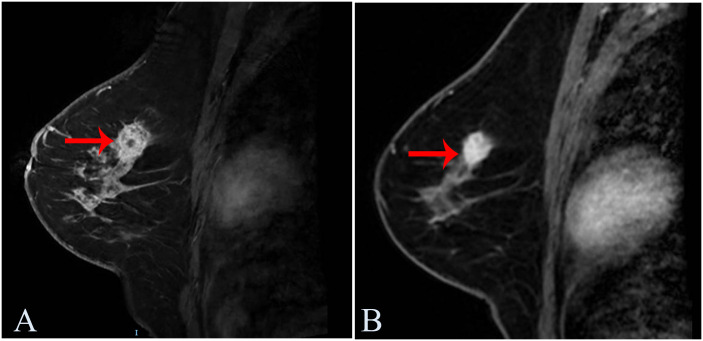
Breast magnetic resonance imaging (MRI). **(A)** Pre-treatment MRI (January 2025): Irregular spiculated mass in the left breast upper outer quadrant, with indistinct margins; lesion size: 1.5 cm × 1.7 cm × 2.1 cm. **(B)** Post-neoadjuvant chemotherapy MRI (April 2025, eight cycles): Partial response with interval reduction in mass size to 1.1 cm × 1.3 cm × 1.7 cm, confirming treatment efficacy.

### 2.2 Immunohistochemical staining

Tissue specimens were fixed in 10% neutral-buffered formalin, embedded in paraffin, and sectioned at 4 μm. Hematoxylin and eosin (H&E) staining was used for histological evaluation. Representative tumor sections were immunostained with available primary antibodies against estrogen receptor (ER), progesterone receptor (PR), human epidermal growth factor receptor 2 (HER2), cluster of differentiation 117 (CD117), cytokeratin 7 (CK7), CD56, synaptophysin (Syn), chromogranin A (CgA), CK5/6, cancer antigen 153 (CA153), P63, SRY−box transcription factor 10 (SOX10), CK19, insulinoma-associated protein 1 (INSM1), GATA binding protein 3 (GATA3), thyroid transcription factor 1 (TTF-1), caudal-type homeobox transcription factor 2 (CDX2), and Ki−67 (Maixin, Fuzhou, China). Immunohistochemistry was performed using EnVision. Negative and positive controls were included in the immunohistochemical assay.

### 2.3 Pathological diagnosis and follow-up history

Tumor specimen was embedded in a paraffin block and examined. A pathological examination of the breast core needle biopsy revealed the invasive growth of carcinoma cells with abundant cytoplasm, showing morphology similar to that of invasive breast carcinoma, NOS (**Figures 2A, B**). The immunohistochemical results showed negative staining for ER, PR, HER2, and CK5/6. The Ki-67 proliferation index was approximately 60%. Lymph node aspiration biopsy demonstrated metastatic carcinoma. The tumor was classified as TNBC, and neoadjuvant chemotherapy was recommended, followed by consideration of surgical treatment. The adjuvant chemotherapy regimen of TCb-EC combined with camrelizumab is formulated. The neoadjuvant regimen was chosen based on the following rationale: (1) biopsy-pathology-diagnosed TNBC, (2) aggressive biology and high Ki-67 (~60%)—platinum-based chemotherapy (carboplatin) is commonly used in high-grade carcinomas, and (3) addition of camrelizumab (anti-PD-1 antibody): emerging evidence supports immunotherapy in advanced/metastatic TNBC, aiming to enhance anti-tumor immunity. After eight cycles of chemotherapy, the breast mass was slightly reduced (**Figure 1B**), and then a modified radical mastectomy of the unilateral breast was performed.​ Preoperative plain CT scans of the upper abdomen, chest (lungs), neck, and craniocerebrum showed a suspicious nodule in the left breast, slightly enlarged lymph node shadows in the left axilla, and no obvious abnormalities in other parts.

**Figure 2:**
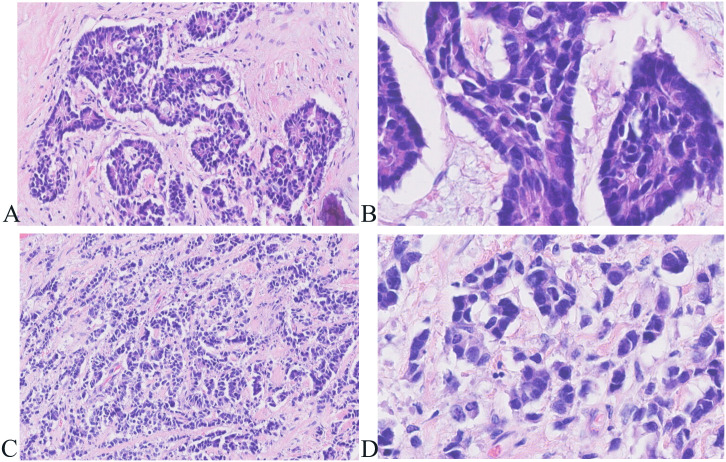
Histopathological features of primary breast LCNEC (H&E staining). **(A, B)** Core needle biopsy. **(A)** Low power (×100): Infiltrative carcinoma with solid nests, abundant eosinophilic cytoplasm, morphologically mimicking invasive breast carcinoma, NOS. **(B)** High power (×400): Large pleomorphic tumor cells, abundant cytoplasm, high nuclear-to-cytoplasmic ratio, coarse chromatin, prominent nucleoli; no glandular differentiation. **(C, D)** Radical resection specimen. **(C)** Low power (×100): Solid/nested architecture of tumor cells, separated by fibrous stroma, with geographic necrosis. **(D)** High power (×400): Uniform large cells with abundant eosinophilic cytoplasm, marked nuclear pleomorphism, irregular nuclear contours, frequent mitoses (>20/10 HPF), consistent with LCNEC.

The patient underwent modified radical mastectomy. One breast was with axillary contents, measuring 24 cm × 20 cm × 4 cm. A firm area was palpable 5 cm inferior to the nipple, measuring 1.8 cm × 1.8 cm × 1.5 cm. The section surface was grayish and hard, and the boundary was unclear. Microscopically, the tumor cells were large and pleomorphic, with abundant eosinophilic cytoplasm, irregular nuclear contours, prominent nucleoli, and frequent mitotic figures (>20 mitoses per 10 high-power fields). The tumors showed solid and nested architecture with geographic necrosis. Lymphovascular invasion was identified. These features were characteristic of LCNEC (**Figures 2C, D**). Immunohistochemically, the tumor cells showed positive expression of CK7, CD117, CD56, CgA, and INSM1 and were focally positive for GATA3, CK19, CA153, SOX10, and Ki-67 (approximately 70% in hot spots), but these were negative for CK5/6, ER, PR, HER2, P63, Syn, TTF-1, and CDX2 (**Figure 3**). Carcinoma metastasis was seen in the lymph nodes (3/14). Tumor response was evaluated using the Miller–Payne pathological grading system for neoadjuvant chemotherapy and was classified as grade 3 (G3), corresponding to a 30%–55% reduction in invasive carcinoma cells.

**Figure 3:**
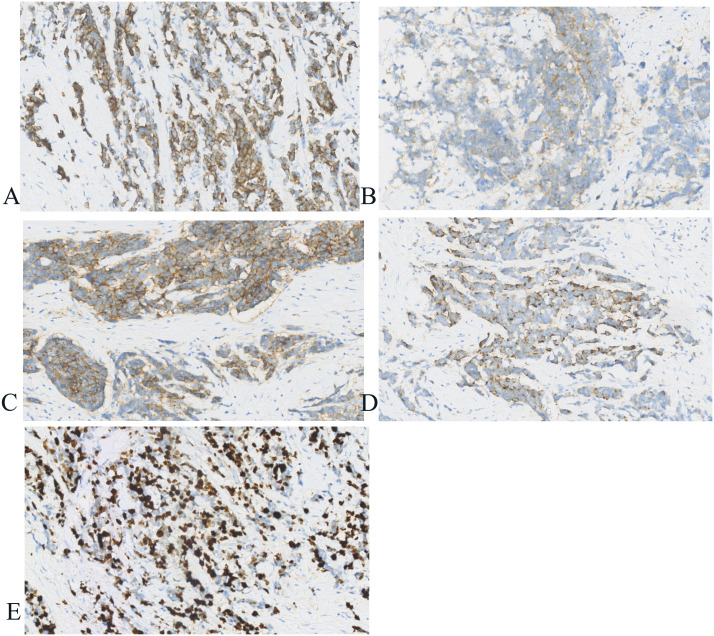
Immunohistochemical staining profile of breast LCNEC (×100). Tumor cells show diffuse positivity for neuroendocrine and epithelial markers: **(A)** CK7 (epithelial origin, positive), **(B)** CD117 (neuroendocrine marker, positive), **(C)** CD56 (neuroendocrine marker, positive), **(D)** CgA (neuroendocrine marker, positive), and **(E)** Ki-67 proliferation index (70%, hot spot, high proliferative activity).

The patient’s final diagnosis was LCNEC of the breast, ypT1cN1aM0, according to the eighth edition of the tumor node metastasis (TNM) staging system, and she was successfully discharged from the hospital after 5 days. Postoperative adjuvant capecitabine was administered because of high-risk features: lymph node metastasis (3/14), triple-negative phenotype, high-grade NEC, and elevated Ki-67 (70%). Oral capecitabine is a well-tolerated adjuvant option in TNBC and extra-pulmonary NEC, which is intended to reduce recurrence risk. The patient received capecitabine chemotherapy after surgery and recovered well. Follow-up was limited to 9 months, and no evidence of recurrence or distant metastasis has been observed so far. However, given the short follow-up duration, firm conclusions regarding long-term efficacy and prognosis cannot be drawn.

## 3 Discussion

Primary NEC of the breast represents an often underdiagnosed subset of breast malignancies, comprising small cell (SCNEC) and LCNEC (9, 10). Due to their rarity, a precise diagnosis remains clinically challenging. Its atypical clinical manifestations and overlapping morphological features with other common breast cancers often lead to misdiagnosis in preoperative biopsy, which further affects the selection of treatment and prognosis evaluation (4, 11–13). This case is reported to discuss the clinical and pathological characteristics, diagnostic dilemmas, and treatment strategies of primary breast LCNEC, providing reference for clinical practice.

Preoperative core needle biopsy is the main method for the pathological diagnosis of breast cancer, but it has certain limitations in the diagnosis of rare breast cancer subtypes (14–17). Breast LCNEC can closely resemble poorly differentiated invasive ductal carcinoma, invasive lobular carcinoma, or solid-type ductal carcinoma *in situ*. Therefore, a precise diagnosis relies on careful morphological assessment, followed by confirmatory IHC analysis (18, 19). In this case, the preoperative biopsy result was invasive breast carcinoma, NOS. The main reason for the misdiagnosis was that the small tissue sample obtained by biopsy failed to show the typical pathological features of LCNEC, and the IHC tests were incomplete.

Modified radical mastectomy was performed after neoadjuvant therapy in this patient, and the postoperative IHC results confirmed the diagnosis of primary breast LCNEC. Consistent with WHO criteria, the diagnosis was established based on large cell morphology, diffuse expression of neuroendocrine markers (CD56, CgA, and INSM1), and exclusion of metastatic disease. Notably, Syn negativity does not exclude LCNEC, as up to 30% of cases lack Syn expression while other neuroendocrine markers are positive. GATA3 and SOX10 positivity confirmed mammary origin, while TTF-1 and CDX2 negativity excluded pulmonary and gastrointestinal metastasis, respectively (7, 19, 20).

Beyond immunohistochemistry, molecular profiling has advanced our understanding of extra-pulmonary neuroendocrine carcinomas (EPNECs). Transcription factor-based subtyping (ASCL1, NEUROD1, INSM1, and YAP1) provides a unified molecular framework across EPNECs. Genomically, breast LCNEC frequently harbors TP53/RB1 alterations and PI3K/AKT/mTOR pathway activation. Metovic et al. (2022) defined INSM1-high and INSM1-low molecular subtypes of EPNECs, with INSM1-low cases associated with poorer prognosis (17, 21). Our case’s diffuse INSM1 positivity aligns with the favorable INSM1-high subtype (21). Given the rarity of breast LCNEC, molecular subtyping improves diagnostic accuracy and prognostic stratification.

Differential diagnosis between SCNEC and LCNEC of the breast is mainly based on histomorphology. SCNEC shows small, monotonous cells with scant cytoplasm, nuclear molding, and high mitotic activity. In contrast, LCNEC consists of large pleomorphic cells with abundant cytoplasm and prominent nucleoli and lacks distinct neuroendocrine differentiation (16, 22). Distinguishing primary breast LCNEC from TNBC with neuroendocrine differentiation and metastatic NEC is critical (16, 20). Primary breast LCNEC exhibits large cell morphology, diffuse neuroendocrine marker expression (INSM1, CgA, and CD56). TTF−1/CDX2 negativity excludes pulmonary/gastrointestinal metastasis. Systemic imaging further ruled out metastatic NEC. TNBC with neuroendocrine differentiation lacks diffuse neuroendocrine expression and typical LCNEC morphology (16, 20, 23). In this case, the postoperative IHC and no history of tumors in other organs clarified the pathological subtype, laying a foundation for subsequent adjuvant treatment and prognosis evaluation.

To the best of our knowledge, including the present case, only 10 cases of primary breast LCNEC have been reported in the English literature to date (3, 6, 22, 24–26). The clinicopathological features of these patients are summarized in **Table 1**. The age at diagnosis ranges from 27 to 85 years, with a mean age of 55.1 years. Tumor size varies from 1.7 to 9.5 cm in greatest dimension, with a mean diameter of 4.5 cm. Lymph node metastasis was documented in two cases, absent in three cases, and unknown in five cases. Immunohistochemically, seven of eight evaluable cases were HER2−negative (score 0). Notably, the present case is the first and only reported triple−negative breast LCNEC (ER−/PR−/HER2−). This immunophenotype is extremely rare for breast LCNEC, which typically exhibits hormone receptor positivity rather than a triple−negative profile resembling conventional TNBC. The unique immunoprofile of our case expands the known phenotypic spectrum of breast LCNEC and highlights potential molecular heterogeneity within this rare tumor subtype. Further clinical and molecular studies are required to clarify its biological behavior, prognostic implications, and optimal therapeutic strategies.

**Table 1:** Clinicopathological characteristics of patients with primary breast large cell neuroendocrine carcinoma.

Case	Year	Age	location	Size (cm)	Lymph node metastasis	Intrinsic subtype	Ki-67 index	Treatment	Follow-up
1 (25)	2011	46	L	3.8	NA	NA	NA	Breast−conserving surgery, adjuvant chemotherapy	6 months without recurrence
2 (24)	2015	34	L	6.0	With	NA	NA	Neo-adjuvant chemotherapy, surgical resection	4 years without recurrence
3 (3)	2024	59	R	2.5	Without	ER (30%+), PR (-), HER2 (0)	40%	Breast−conserving surgery, adjuvant chemotherapy, radiotherapy, endocrine therapy	NA
4 (6)	2024	67	R	2.0	Without	ER (95%, strong+), PR (40%, medium-strong+), HER2 (0)	40%	Breast−conserving surgery, adjuvant chemotherapy, radiotherapy, endocrine therapy	NA
5 (22)	2025	85	R	3.0	NA	ER (90%+), PR (40%+), HER2 (0)	25%	NA	NA
6 (22)	2025	73	R	1.7	Without	ER (95%+), PR (5%+), HER2 (0)	80%	Total mastectomy, endocrine therapy	2.5 years without recurrence
7 (22)	2025	27	NA	6.8	NA	ER (90%+), PR (20%+), HER2 (0)	20%	Surgical resection, endocrine therapy	NA
8 (22)	2025	49	L	9.5	NA	ER (85%+), PR (5%+), HER2 (0)	40%	Palliative therapy	Died at 1 month later
9 (26)	2025	55	L	3.1	With	ER (−), PR (−), HER2 (+)	NA	Partial mastectomy	Lost to follow-up
10 (present case)	2026	47	L	4.5	With	ER (-), PR (-), HER2 (0)	70%	Neo-adjuvant therapy, total mastectomy, postoperative adjuvant capecitabine	9 months without recurrence

NA, not available; R, right; L, left; ER, estrogen receptor; PR, progesterone receptor; HER2, human epidermal growth factor receptor 2.

At present, there is no standardized treatment regimen for LCNEC of the breast, and patients are generally managed with conventional breast cancer therapies (27, 28). LCNEC tends to exhibit a more aggressive behavior than invasive ductal carcinoma, characterized by higher rates of local and distant recurrence as well as inferior overall survival (5). The limitations of this report include the short follow-up (9 months), small sample size, and lack of molecular profiling. Longer follow-up and larger series are required to establish robust conclusions regarding treatment efficacy and prognosis.

## 4 Conclusions

In summary, we present a rare case of primary LCNEC of the breast supported by typical large cell morphology, detailed immunohistochemical results, and a comprehensive PET/CT scan. Complete surgical resection remains the optimal therapeutic approach. Thorough evaluation of histologic features combined with immunohistochemistry is essential to establish an accurate diagnosis. Neoadjuvant chemotherapy with platinum and anti-PD-1 immunotherapy followed by adjuvant capecitabine achieved short-term disease control in this case; however, long-term outcomes require prolonged observation.

## Data Availability

The original contributions presented in the study are included in the article/supplementary material. Further inquiries can be directed to the corresponding author.
